# Research progress on sagittal balance and minimal clinically important difference after two-level lumbar spondylolisthesis surgery

**DOI:** 10.3389/fsurg.2026.1912779

**Published:** 2026-09-01

**Authors:** Xiangyu Zhang, Bin Li, Kai Hu, Jinhong He, Haishan Guan

**Affiliations:** 1 The Second Clinical Medical College, Shanxi Medical University, Taiyuan, Shanxi, China; 2 Department of Spine Surgery, The Second Hospital of Shanxi Medical University, Taiyuan, Shanxi, China

**Keywords:** lumbar fusion, lumbar lordosis, minimal clinically important difference, sagittal alignment, spinopelvic parameters, two-level lumbar spondylolisthesis

## Abstract

Sagittal alignment influences functional recovery after surgery for two-level lumbar spondylolisthesis, yet evidence specific to this population remains limited. This review synthesizes current findings on postoperative changes in lumbar lordosis (LL), sagittal vertical axis (SVA), pelvic tilt, and pelvic incidence–lumbar lordosis mismatch (PI–LL), and examines their associations with patient-reported outcomes. Available studies indicate that two-level fusion generally produces greater LL correction than single-level fusion, although restoration of global sagittal balance and ideal PI–LL matching is less consistent. Improvements in LL and PI–LL are associated with better Oswestry Disability Index (ODI) and pain outcomes, and reported thresholds such as LL improvement of at least 8° and postoperative PI–LL of 10° or less may help predict achievement of the ODI minimal clinically important difference (MCID). These values should be interpreted as radiographic predictors of clinically meaningful improvement rather than MCID thresholds for the radiographic parameters themselves. Current evidence is constrained by heterogeneous study designs, small retrospective cohorts, inconsistent definitions, and limited two-level subgroup analyses. Prospective multicentre studies using standardized radiographic assessment, validated patient-reported anchors, and stratification by age, fusion level, and pelvic morphology are needed to define reliable surgical correction targets.

## 1 Introduction

Degenerative lumbar spondylolisthesis is characterized by anterior vertebral translation associated with progressive degeneration of the intervertebral disc, facet joints, and posterior ligamentous complex, which can produce segmental instability before later restabilization (1). Paraspinal muscular degeneration may also contribute to disease progression, as greater multifidus and erector spinae fatty infiltration has been associated with increased vertebral slippage (2). The resulting structural and neural changes commonly present as lumbar spinal stenosis, mechanical instability, radiculopathy, axial pain, and functional limitation. Sagittal assessment is particularly relevant because degenerative spondylolisthesis may alter both total lumbar lordosis and its distribution across the lower lumbar segments (3). When degeneration and vertebral translation involve two lumbar levels, surgical planning becomes more complex because decompression and fusion modify motion across a larger portion of the lumbar spine and increase dependence on the remaining mobile segments and pelvic compensatory mechanisms (4). Two-level lumbar spondylolisthesis therefore requires evaluation of local neural decompression, segmental correction, regional lordosis distribution, and global spinopelvic alignment.

Sagittal alignment is commonly assessed using lumbar lordosis (LL), segmental lumbar lordosis (SLL), sagittal vertical axis (SVA), pelvic incidence (PI), pelvic tilt (PT), sacral slope (SS), and pelvic incidence–lumbar lordosis mismatch (PI–LL). LL describes the regional lumbar curvature, whereas SLL quantifies lordosis across the treated motion segments. SVA reflects anterior displacement of the trunk, PT represents pelvic retroversion used to compensate for forward imbalance, and PI–LL expresses the correspondence between fixed pelvic morphology and lumbar curvature (5). Degenerative lumbar spondylolisthesis may coexist with lower-lumbar hypolordosis and compensatory hyperextension at the affected level, indicating that apparently normal total LL can conceal an abnormal distribution of lordosis (3). Higher PI has also been associated with greater SVA and more advanced sagittal malalignment in low-grade degenerative spondylolisthesis (6). These observations support preoperative assessment of the distribution and proportionality of lordosis in addition to absolute LL values (7). Paraspinal muscle integrity is also relevant because reduced muscle mass and greater fatty infiltration have been associated with poorer maintenance of sagittal balance after multilevel lumbar fusion (8).

The association between sagittal alignment and health-related quality of life was initially established in adult spinal deformity populations. Lafage et al. (9) identified PT and truncal inclination as clinically relevant measures of compensatory pelvic rotation and anterior trunk displacement. Schwab et al. (10) subsequently demonstrated that increasing PI–LL mismatch, PT, and SVA were associated with progressively greater disability, providing the radiographic basis for commonly used sagittal severity thresholds. The Scoliosis Research Society–Schwab classification formalized PI–LL, PT, and SVA as sagittal modifiers associated with health-related quality of life and treatment selection (11). These modifiers provide an established framework for describing malalignment, although their thresholds were developed primarily in adult spinal deformity cohorts and cannot be transferred directly to short-segment degenerative fusion without population-specific validation.

Additional alignment models have expanded assessment beyond isolated linear measurements. Protopsaltis et al. (12) introduced the T1 pelvic angle (T1PA), which combines trunk inclination and pelvic retroversion in a single angular measure and correlates with the Oswestry Disability Index (ODI) and other health-related quality-of-life measures. The Global Alignment and Proportion (GAP) score evaluates relative pelvic version, relative LL, lordosis distribution, relative spinopelvic alignment, and age to identify proportional or disproportional alignment (13). These frameworks emphasize that alignment should be interpreted relative to pelvic morphology, age, and the distribution of lumbar curvature. Their principal validation populations involved extensive adult spinal deformity correction, however, and their relevance to two-level lumbar spondylolisthesis should be considered supportive rather than definitive.

Patient-reported outcomes remain essential for determining whether radiographic correction produces meaningful clinical benefit. Contemporary evidence in degenerative lumbar spondylolisthesis indicates that patients achieving the ODI minimal clinically important difference (MCID) after fusion may demonstrate greater improvements in SLL, SS, PT, and PI–LL. Higher change in SLL, higher change in SS, and higher postoperative SS have been identified as independent radiographic predictors of MCID achievement (14). These findings define an association between sagittal correction and patient-reported improvement; they do not establish an MCID for SLL, LL, SVA, SS, or PI–LL. The distinction is particularly important because the available predictive evidence predominantly concerns single-level degenerative lumbar spondylolisthesis, whereas two-level disease involves a larger fused region, different lordosis-distribution requirements, and reduced postoperative compensatory capacity.

The MCID was originally defined as the smallest difference in an outcome that patients perceive as beneficial and that could justify a change in management after consideration of treatment risks and costs (15). Subsequent low-back-pain research operationalized clinically important change by comparing questionnaire-score changes with patient-reported global ratings of improvement (16). In spinal research, MCID therefore applies primarily to validated patient-reported outcome measures such as the ODI, visual analogue scale (VAS), Short Form health surveys, and Scoliosis Research Society questionnaires (17). Anchor-based methods linked to patient-perceived change remain the preferred approach, while distribution-based estimates principally identify change exceeding statistical variability or measurement error (18). A radiographic change can be described as a predictor of achieving MCID, a radiographic correction threshold, or a surgical alignment target when it discriminates between patients who do and do not achieve meaningful improvement. It should not be designated as an independent radiographic MCID without a validated patient-perception, functional, or prognostic anchor.

Evidence specific to two-level lumbar spondylolisthesis remains limited by small retrospective cohorts, inconsistent disease definitions, mixed inclusion of degenerative and isthmic pathology, variation in fusion approaches, and insufficient stratification by operated level. A recent meta-analysis identified only 10 comparative studies of single-, double-, and multilevel lumbar fusion and rated the certainty of the pooled evidence as low to very low, confirming the limited and heterogeneous evidence available for multilevel constructs (19). A multicentre study of one- and two-level fusion for degenerative lumbar disease further demonstrated the importance of matching total and segmental lumbar lordosis to pelvic morphology, although the combined analysis did not provide isolated estimates for two-level spondylolisthesis (20). Alignment thresholds derived from adult spinal deformity may therefore overstate the correction required for short-segment fusion, whereas thresholds derived from single-level disease may not represent the altered lordosis distribution and compensatory demands of two-level constructs. Dynamic radiographic assessment has also shown that pelvic incidence significantly influences changes in pelvic tilt, sacral slope, total lumbar lordosis, and lower lumbar lordosis between standing and sitting positions (21). These findings support evaluation of pelvic morphology, baseline sagittal alignment, postural compensatory capacity, and fusion extent when interpreting radiographic predictors and clinical outcomes after two-level fusion.

The study seeks to (i) characterize changes in regional and global sagittal alignment after surgery for two-level lumbar spondylolisthesis; (ii) examine associations between LL, SLL, SVA, PT, SS, PI–LL, and validated patient-reported outcomes; (iii) determine the predictive value of sagittal correction for achieving MCID in ODI, VAS, and related clinical measures; and (iv) compare single-level and two-level fusion across operated levels and surgical approaches to identify radiographic predictors of clinically meaningful recovery and potential surgical alignment targets.

## 2 Methods

The study was conducted and reported in accordance with the Preferred Reporting Items for Systematic Reviews and Meta-Analyses 2020 (PRISMA 2020) framework. The methods included predefined eligibility criteria, systematic database searching, duplicate removal, independent screening, standardized data extraction, design-specific quality appraisal, and structured narrative synthesis.

### 2.1 Eligibility criteria

#### 2.1.1 Inclusion criteria

Studies were eligible when they met the following criteria: (i) adults aged ≥18 years with radiographically confirmed degenerative or isthmic spondylolisthesis involving two lumbar motion segments; (ii) decompression with instrumented PLIF, TLIF, OLIF, ALIF, LLIF, posterolateral fusion, or a combined approach; (iii) comparison with single-level fusion, another two-level technique, alternative cage placement or lordosis, preoperative status, sagittal-balance category, or MCID-achievement group, where applicable; (iv) reporting of at least one radiographic outcome, including LL, SLL, SVA, PI, PT, SS, PI–LL, T1PA, GAP score, disc height, slip percentage, or alignment correction; (v) reporting of at least one clinical outcome, including ODI, VAS, Short Form scores, SRS scores, JOA scores, satisfaction, reoperation, adjacent-segment degeneration, or MCID achievement; (vi) randomized studies, comparative studies, cohort studies, case-control studies, cross-sectional radiographic studies, systematic reviews, or meta-analyses; (vii) case series with ≥10 two-level cases and extractable outcomes; and (viii) English- or Chinese-language publication between 1 January 2010 and 31 March 2026. Pre-2010 studies were retained only when they established an MCID definition, validated a sagittal parameter, or introduced a widely used alignment classification. Studies enrolling broader lumbar populations were eligible only when two-level findings were separately extractable or the study evaluated an alignment construct directly applicable to two-level fusion (Table 1).

**Table 1:** Sagittal correction after two-level fusion.

First author	Year	Population or comparison	Parameters	Principal finding
Wei Yangyang et al. (22)	2020	Single-level vs. two-level PLIF	LL, PI–LL	Two-level fusion produced approximately 5°–8° greater LL correction but less frequent PI–LL matching within ±10°
Zhang Wenhong et al. (23)	2023	Double-segment PLIF, *n* = 95	SSA, TPA, LL, PI, PT, SS	Sagittal changes correlated with VAS and ODI improvement; combined AUC = 0.927
Du et al. (24)	2019	Single-level vs. two-level TLIF	SLL, global alignment	Two-level fusion improved SLL without superior global balance
Zeng Xiantie et al. (25)	2013	Two- to three-level fusion, *n* = 72	LL, SVA	Each additional level increased LL correction by approximately 2.3° but reduced SVA improvement by about 8%
Li et al. (27)	2024	MRI muscle analysis, *n* = 140	SVA, PI–LL, muscle fatty infiltration	Greater paraspinal fatty infiltration was associated with worse sagittal alignment
Li Changkuan et al. (28)	—	Two-level MIS-TLIF	LL	LL increased from 41.8° ± 9.2° to 48.5° ± 7.6°
Hasegawa et al. (64)	2020	Single-level vs. two-level disease	PI, compensatory pattern	Two-level disease showed greater pelvic compensation and postoperative decompensation risk

#### 2.1.2 Exclusion criteria

**Figure 1:**
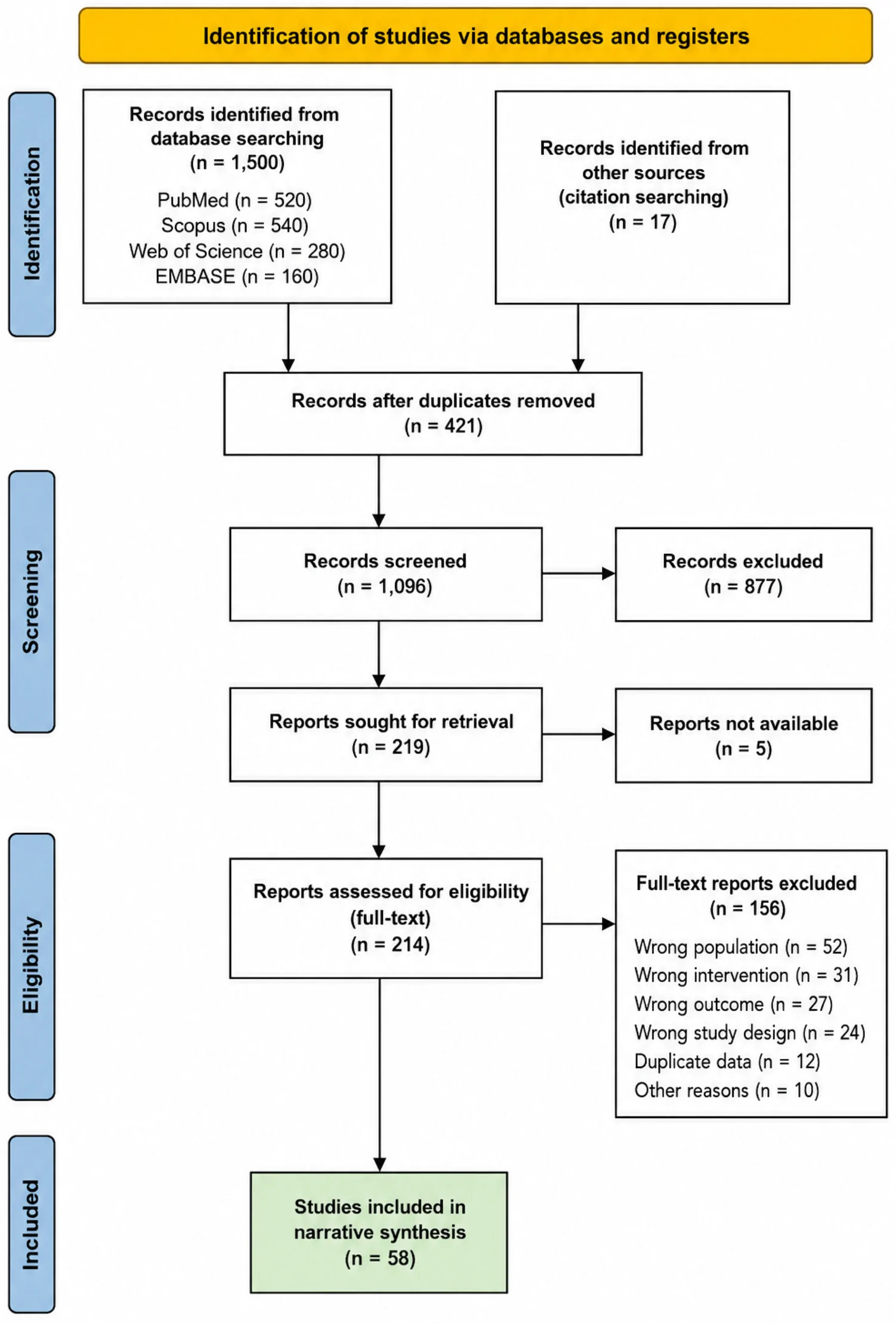
PRISMA 2020 flow diagram of study identification and selection**.** Database searches identified 1,500 records and citation searching identified 17 additional records. After removal of 421 duplicates, 1,096 records were screened, 877 were excluded, 219 reports were sought, five were unavailable, and 214 full texts were assessed. Full-text assessment excluded 156 reports, leaving 58 articles for narrative synthesis.

Studies were excluded when they met any of the following criteria: (i) single-level disease without a two-level comparator or separable subgroup; (ii) cervical, thoracic, traumatic, neoplastic, infectious, or exclusively high-grade congenital pathology; (iii) decompression without fusion; (iv) absence of sagittal, patient-reported, or MCID-related outcomes; (v) cadaveric, animal, finite-element, or purely biomechanical design; (vi) technical notes, conference abstracts, editorials, letters, or non-systematic opinion articles; (vii) unavailable full text or non-extractable outcomes; or (viii) duplicate or overlapping cohorts. Overlapping reports were compared by institution, recruitment period, sample size, and baseline characteristics. The report with the largest sample, longest follow-up, or most complete outcomes was retained; complementary reports contributed only non-duplicated data. Country, journal origin, and database indexing were not exclusion criteria (Figure 1, Supplementary Table 1).

### 2.2 Information sources and search strategy

The final search was completed on 31 March 2026. Sources included PubMed/MEDLINE, Embase, Scopus, Web of Science Core Collection, the Cochrane Library, ScienceDirect, SpringerLink, CNKI, Wanfang Data, and the VIP Chinese Science and Technology Journal Database. Dissertations were searched through ProQuest Dissertations and Theses, CNKI, and Wanfang. Reference lists of included studies and major reviews were screened manually. Forward citation searches were conducted in Scopus and Google Scholar. Pre-2010 foundational studies identified through citation searching were assessed using the same eligibility criteria, except for the publication-date restriction. The strategy combined four concepts: lumbar spondylolisthesis, lumbar fusion, sagittal alignment, and clinically meaningful outcomes. Controlled vocabulary and free-text terms were searched in titles, abstracts, keywords, and subject headings. Two-level terms were included without restricting retrieval because relevant subgroups were frequently embedded within broader fusion cohorts (Table 2 and Supplementary Table 2).

**Table 2:** Sagittal parameters and clinical outcomes.

First author	Year	Population	Parameters	Outcomes	Principal finding
Glassman et al. (30)	2005	Post-fusion patients, *n* = 298	SVA	ODI	ODI increased by approximately 2.5 points per 10 mm increase in SVA
Liu Changhao et al. (31)	2025	DLS, *n* = 89; two-level *n* = 32	LL	ODI, VAS	LL improvement correlated with ODI improvement (r = 0.62, *p* < 0.01)
Sun et al. (32)	2020	Post-OLIF, *n* = 65	LL, SVA	ODI, VAS	Target LL restoration was associated with a 32% higher ODI improvement rate
Zhang Wenhong et al. (23)	2023	Double-segment PLIF, *n* = 95	SSA, TPA, LL, PT, SS	ODI, VAS	Greater sagittal change correlated with greater functional and pain improvement
Korsun et al. (33)	2024	Lumbar spondylolisthesis, *n* = 59	PI–LL, SVA	ODI, VAS	Lower PI–LL and SVA were associated with improvement, although clinical–radiographic discordance remained
Cai et al. (34)	2024	Degenerative and isthmic disease	SK, PI–LL	ODI	Higher SK was associated with poorer PI–LL matching and higher ODI
Elmorsy et al. (35)	2024	Individualized vs. routine planning	LL target	ODI	Individualized planning increased the one-year ODI improvement rate by 22%
Radovanovic et al. (37)	2017	Degenerative lumbar spondylolisthesis after decompression and fusion, *n* = 84; SVA <50 mm, 46.4%; SVA ≥50 mm, 53.6%	SVA, LL, PI–LL	ODI, SF-36 PCS, back pain	The SVA ≥50 mm group had worse ODI (*p* = 0.043), SF-36 PCS (*p* = 0.018), and back pain (*p* = 0.039). LL was lower (49.8° ± 4.3° vs. 56.4° ± 4.7°, *p* = 0.040), and PI–LL matching within ±9° was less frequent (23.1% vs. 51.0%, *p* = 0.013)

### 2.3 Study selection

#### 2.3.1 Screening process

Database searches identified 1,500 records, including 1,389 English-language and 111 Chinese-language records. Citation searching added 17 records, yielding 1,517 records. Removal of 421 duplicates left 1,096 records for title and abstract screening. Two reviewers screened all records independently. Records considered potentially eligible by either reviewer proceeded to full-text review. Title and abstract screening excluded 877 records. Of 219 reports sought, five were unavailable and 214 underwent full-text assessment. A further 156 reports were excluded, leaving 58 included articles: 44 primary observational studies, eight systematic reviews or meta-analyses, and six foundational methodological or classification studies. Foundational studies informed definitions, measurement constructs, and alignment classifications but were excluded from treatment-effect conclusions.

#### 2.3.2 Consensus and software

References were deduplicated using EndNote 21 (Clarivate, United States). Screening was conducted using the Rayyan web version, accessed March 2026 (Rayyan Systems Inc., United States), with reviewer decisions blinded until conflict resolution. Disagreements were resolved by discussion, followed by third-reviewer adjudication when required. Cohen’s K was calculated before consensus, with K ≥ 0.80 indicating strong agreement (Supplementary Table 3).

### 2.4 Data extraction

#### 2.4.1 Extraction mechanics

A standardized form was developed in Microsoft Excel 365 (Microsoft Corporation, United States) and pilot-tested on six studies representing different designs and languages. Two reviewers extracted data independently and compared entries field by field. Disagreements were resolved against the full text, with third-reviewer adjudication when required. Angular values were recorded in degrees and linear values in millimetres. Means and standard deviations were extracted where available; medians and interquartile ranges were retained as reported. Figure-only values were not digitized. Missing variables were coded as “not reported,” and no data were imputed.

#### 2.4.2 Extracted data items

Extracted variables comprised study design, setting, population, pathology, operated levels, surgical approach, follow-up, sagittal parameters, patient-reported outcomes, MCID method, MCID threshold, achievement rate, predictor estimates, and quality indicators. Radiographic correction targets were recorded separately from patient-anchored MCID estimates (Supplementary Table 4).

### 2.5 Quality assessment

#### 2.5.1 Appraisal tools

Cohort and case-control studies were assessed using the Newcastle–Ottawa Scale (NOS; Universities of Newcastle and Ottawa, Australia and Canada). The NOS evaluated: (i) study-group selection; (ii) group comparability; and (iii) outcome or exposure ascertainment. Scores of 7–9 indicated low risk of bias, 5–6 moderate risk, and 0–4 high risk. Systematic reviews retained as secondary evidence were assessed using A Measurement Tool to Assess Systematic Reviews 2 (AMSTAR 2; University of Ottawa, Canada). AMSTAR 2 evaluated protocol registration, search adequacy, study selection, data extraction, risk-of-bias assessment, synthesis methods, publication bias, and conflicts of interest. Overall confidence was classified as high, moderate, low, or critically low. Review-level bias was assessed using the Risk of Bias in Systematic Reviews (ROBIS; University of Bristol, United Kingdom). ROBIS covered: (i) eligibility criteria; (ii) study identification and selection; (iii) data collection and appraisal; and (iv) synthesis and interpretation. Overall judgments were low, high, or unclear risk of bias. Foundational methodological and classification studies were appraised for: (i) construct clarity; (ii) derivation or validation sample adequacy; (iii) measurement reproducibility; and (iv) external validation or adoption. These studies informed definitions and conceptual frameworks and were not assigned treatment-effect scores.

#### 2.5.2 Assessment procedure

Two reviewers completed all assessments independently. Disagreements were resolved through full-text review and third-reviewer adjudication. Ratings from different tools were not combined. Moderate-risk studies remained eligible. High-risk observational studies and critically low-confidence reviews were used only as contextual evidence and did not independently support numerical thresholds or clinical recommendations. Greater weight was assigned to multicentre, prospective, adequately adjusted, longer-term, and low-risk evidence (Supplementary Table 5). Journal location, language, and indexing status were not treated as proxies for methodological quality. Regional studies underwent the same design-specific appraisal as all other evidence, and their contribution was determined by NOS, AMSTAR 2, or ROBIS ratings, sample adequacy, outcome reporting, confounder control, and follow-up completeness. Seven of the 58 included articles (12.1%) were published in regional Chinese journals; these studies were retained for population-specific evidence but were not used independently to establish MCID predictors or universal alignment thresholds.

### 2.6 Data synthesis

#### 2.6.1 Narrative synthesis

Differences in pathology, fusion level, surgical approach, parameter definitions, follow-up, MCID methods, and covariate adjustment precluded overall meta-analysis. A structured narrative synthesis was therefore conducted. Studies were grouped by: (i) single-level, two-level, or multilevel fusion; (ii) degenerative or isthmic pathology; (iii) posterior, anterior, lateral, or oblique approach; (iv) L3–L4/L4–L5, L4–L5/L5–S1, or other segment combinations; (v) follow-up of <12, 12–24, or >24 months; and (vi) regional alignment, global balance, PI–LL matching, patient-reported improvement, MCID achievement, or mechanical outcomes. Sagittal variables associated with patient-reported MCID achievement were classified as radiographic predictors. Values derived through regression, receiver-operating-characteristic analysis, or group comparison were classified as predictive thresholds or alignment targets, not independent radiographic MCID values.

#### 2.6.2 Heterogeneity and missing data

Heterogeneity was evaluated across population, pathology, operated levels, approach, measurement method, follow-up, MCID definition, and risk of bias. Findings were compared only when definitions and assessment times were compatible. Missing data were reported without imputation. Conflicting findings were weighted according to two-level sample size, prospective or multicentre design, standardized standing radiographs, confounder adjustment, follow-up duration, and risk of bias. Single-level and adult spinal deformity studies were retained only as contextual evidence.

## 3 Synthesis of findings

The 58 included articles comprised 44 observational studies, eight systematic reviews, and six foundational methodological or classification studies. The evidence was synthesized across postoperative sagittal correction, associations with clinical outcomes, radiographic prediction of MCID achievement, modifying clinical and surgical factors, and limitations affecting interpretation. Direct evidence from two-level spondylolisthesis was prioritized, while single-level, multilevel, and adult spinal deformity studies were used as contextual evidence.

### 3.1 Sagittal correction

Two-level fusion generally produced greater local lordotic correction than single-level fusion, although improvement in global sagittal balance was less consistent. Wei Yangyang et al. (22), reported approximately 5°–8° greater LL correction after two-level PLIF, but fewer patients achieved PI–LL within ±10°. This pattern indicates that increasing correction across the fused segments does not ensure proportional restoration of whole-spine alignment.

Zhang Wenhong et al. (23), evaluated 95 patients with double-segment lumbar spondylolisthesis treated by PLIF. Postoperative increases in SSA, TPA, LL, and SS and reductions in PT were associated with greater improvements in VAS and ODI at three months. The combined sagittal parameters achieved an AUC of 0.927 for discriminating good from poor clinical recovery, with 90.50% sensitivity and 91.89% specificity. These findings support multidimensional assessment because no single parameter fully represented postoperative recovery.

Du et al. (24), found that two-level TLIF improved SLL more than single-level TLIF without a corresponding advantage in global alignment. Zeng Xiantie et al. (25), similarly reported that each additional fused level increased LL correction by approximately 2.3°, while SVA improvement declined by about 8%. These results suggest that longer constructs increase regional correction but restrict residual lumbar mobility and pelvic compensation.

Anatomical heterogeneity further affected alignment. Zhang et al. (26), associated more sagittally oriented facet joints with reduced shear resistance and greater instability. Li et al. (27), examined paraspinal muscle morphology by magnetic resonance imaging and linked greater multifidus and erector spinae fatty infiltration to higher SVA and PI–LL. Li et al. (27) therefore represents a muscle-imaging study and should not be attributed to MIS-TLIF. The MIS-TLIF findings belong to Li Changkuan et al. (28), who reported an increase in LL from 41.8° ± 9.2° to 48.5° ± 7.6°.

Segment location also influenced compensatory patterns. Lyu et al. (29), found that L4–L5/L5–S1 disease was associated with higher PI, PT, and SVA, whereas L3–L4/L4–L5 disease showed higher SS and LL and lower SVA. Lower-lumbar involvement therefore appears to impose greater pelvic compensation and global imbalance. This level-specific variation limits the validity of applying a single correction target to all two-level constructs.

### 3.2 Clinical outcomes

Improved sagittal alignment was generally associated with lower disability and pain, but the relationship was neither uniform nor strictly linear. Glassman et al. (30), reported an average 2.5-point increase in ODI for each 10 mm increase in SVA among 298 patients. Liu Changhao et al. (31), found that LL improvement correlated with ODI improvement in degenerative spondylolisthesis, with a stronger association in the two-level subgroup (r = 0.62, *p* < 0.01).

Sun et al. (32), reported a 32% higher ODI improvement rate among patients whose LL reached the defined postoperative range after OLIF. Korsun et al. (33), found that each 1° reduction in PI–LL corresponded to a 0.8-point reduction in ODI and each 10 mm reduction in SVA to a 0.6-point reduction in VAS back pain. However, the same cohort showed substantial ODI and VAS improvement despite decreased mean LL and increased SVA. This clinical–radiographic discordance indicates that absolute alignment values may inadequately represent symptom recovery when baseline anatomy, compensatory capacity, and patient-specific targets are ignored.

Additional evidence supports morphology-adjusted interpretation. Cai et al. (34), linked greater SK to worse PI–LL matching and higher ODI. Elmorsy et al. (35), reported a 22% higher one-year ODI improvement rate when individualized LL targets were incorporated into surgical planning. Li X et al. (36), found superior ODI and VAS improvement when postoperative LL approached PI ± 9°. Conversely, Radovanovic et al. (37) found that patients with postoperative SVA ≥50 mm had worse ODI, SF-36 physical-component scores, and back pain than those with SVA <50 mm, indicating that residual global sagittal imbalance may adversely affect patient-reported recovery. Additional evidence supports morphology-adjusted interpretation. Cai et al. (34) linked greater SK to poorer PI–LL matching and higher ODI. Elmorsy et al. (35) reported a 22% higher one-year ODI improvement rate when individualized LL targets informed surgical planning, while Li X et al. (36) found greater ODI and VAS improvement when postoperative LL approached PI ± 9°. These findings indicate that sagittal correction contributes to recovery, although its clinical value depends on baseline morphology, proportional alignment, and patient-specific functional demands.

### 3.3 MCID prediction

MCID applies to changes in patient-reported outcomes that patients perceive as meaningful. Radiographic measures therefore function as predictors of MCID achievement or surgical alignment targets, not independent MCID outcomes.

Alentado et al. (38), found that preoperative SVA >50 mm reduced the probability of achieving ODI MCID by 58%. Wang et al. (14), identified LL improvement ≥8° and postoperative PI–LL ≤10° as predictors of ODI MCID, with sensitivities of 72% and 76% and specificities of 68% and 70%, respectively. These values should be interpreted as discrimination thresholds for patient-reported improvement.

Maayan et al. (39), reported ODI MCID achievement in 56.3% of patients with age-adjusted SVA abnormalities and 78.5% of those with normal alignment (*p* < 0.01). Subsequent multicentre evidence identified preoperative SVA and PT as additional predictors (40). Power et al. (41), derived anchor-based MCID values of 12.8 points for ODI, 2.1 points for VAS leg pain, and 1.9 points for VAS back pain in 2,156 patients. These patient-anchored values provide the appropriate outcomes against which radiographic predictors should be evaluated.

Single parameters remained insufficient. Chisango et al. (42), found that *Δ*L1PA <5° did not exclude meaningful morphological change, supporting combined assessment. Singh et al. (43), identified lower baseline leg-pain VAS, sagittal facet orientation, greater slip percentage, and increased L5–S1 mobility as predictors of delayed or absent MCID achievement. Yang et al. (44), reported 82% accuracy for a random-forest model in which postoperative LL and PI–LL were the most influential predictors. These findings indicate that MCID achievement reflects an interaction between radiographic alignment, baseline symptoms, anatomy, and operative characteristics.

Patient satisfaction also requires separate interpretation. Evidence cited in (45) and (46) indicates that some patients report satisfactory outcomes without meeting a questionnaire-specific MCID. This does not validate radiographic MCID; it demonstrates that MCID, satisfaction, pain, and functional status measure related but distinct outcome domains.

### 3.4 Modifying factors

Preoperative alignment, fusion extent, operative approach, age, bone quality, and muscular condition modified sagittal correction and its clinical relevance. Severe baseline PI–LL mismatch may permit greater absolute correction while reducing the probability of reaching a proportional target (47, 48). Shimamura et al. (49), found that SVA progression ≥50 mm was associated with higher VAS back pain, higher ODI, and lower satisfaction after short-segment fusion.

Approach-specific differences were most consistent for regional correction. Li et al. (50) reported greater LL restoration and closer PI–LL matching after OLIF than TLIF, while Woods et al. (51) attributed this advantage to disc-height restoration and preservation of posterior structures. Lechtholz-Zey et al. (52) found that ALIF and OLIF generally achieved greater SLL and LL restoration than TLIF, although correction varied across techniques and study populations. Kepler et al. (53) showed that endplate integrity and cage position influenced correction after TLIF, whereas Pennington et al. (54) identified preoperative SLL, implant lordosis, PI, and bilateral cage placement as stronger predictors than cage shape. Zhou et al. (55) further linked screw trajectory and dimensions to construct stability.

Fusion extent altered adjacent-level demand and lordosis distribution. Umehara et al. (56), associated increasing fusion length with greater adjacent-segment alignment change. Zhang et al. (57), reported more complex global compensatory responses after two-level surgery than after single-level procedures. These findings support level-specific planning, especially for L3–L4/L4–L5 and L4–L5/L5–S1 constructs.

Age and bone quality further constrain correction. Yamato et al. (58), supported age-adjusted alignment targets because older patients tolerate less correction and have reduced muscular and ligamentous compensation. Tsujimoto et al. (59), linked advanced age and osteoporosis to fixation failure and correction loss. Greater radiographic correction may therefore be inappropriate when mechanical reserve is limited.

Comparative studies generally favoured OLIF for LL, SS, SVA, and slip reduction (60, 61), whereas MIS-TLIF reduced muscular disruption but offered less correction than open procedures (62). Evidence from high-grade disease (63) should remain contextual because its pathology and correction demands differ from low-grade two-level degenerative disease.

### 3.5 Clinical and practical implications

Preoperative planning should integrate SVA, PI, PT, SS, LL, SLL, PI–LL, lordosis distribution, operated levels, age, bone quality, and paraspinal muscle condition. Surgical correction should restore proportional regional and global alignment while preserving sufficient compensatory capacity. Greater LL correction alone should not be treated as evidence of superior recovery because clinical benefit also depends on baseline morphology, neural decompression, fusion extent, and patient-specific functional demands.

Radiographic parameters should be interpreted as predictors of ODI or VAS MCID achievement and as potential alignment targets. Thresholds such as LL improvement ≥8°, postoperative PI–LL ≤10°, and SVA <50 mm may support planning and prognosis, but their application should remain individualized because most were derived from single-level or mixed lumbar populations. Postoperative evaluation should combine sagittal measurements with patient-reported pain, disability, satisfaction, and functional recovery.

Approach selection should reflect the required correction and the patient's mechanical reserve. OLIF and other anterior or lateral approaches may provide greater disc-height and lordosis restoration, whereas posterior approaches may be preferable when direct decompression or posterior stabilization is required. In older patients and those with osteoporosis or paraspinal degeneration, conservative correction targets and enhanced fixation strategies may reduce correction loss and mechanical complications.

### 3.6 Limitations and future research

The evidence base was dominated by small retrospective and single-centre studies. Definitions of two-level disease, segment combinations, fusion techniques, radiographic acquisition, follow-up intervals, and MCID methods varied substantially. Several studies combined single-level, two-level, and broader multilevel cohorts without reporting separate two-level estimates, limiting direct inference.

Most proposed radiographic thresholds were derived from single-level degenerative disease or adult spinal deformity cohorts. Their transfer to two-level fusion remains uncertain because two-level constructs alter lordosis distribution, residual mobility, and pelvic compensation. Residual confounding was also likely because baseline deformity, cage position, surgeon technique, rehabilitation, bone quality, and muscle degeneration were inconsistently controlled. Regional Chinese studies represented 12.1% of the included evidence and contributed clinically relevant data on local populations and operative practice. Their influence on the synthesis was limited by design-specific quality ratings, and numerical thresholds were supported only when consistent with stronger prospective, multicentre, or internationally indexed evidence. This weighting reduced dependence on journal provenance while preventing methodologically weak regional evidence from driving clinical recommendations.

Future research should prioritize multicentre prospective cohorts with standardized definitions of two-level spondylolisthesis, separate reporting by segment combination, standing full-spine radiographs, predefined follow-up intervals, and multivariable adjustment. Anchor-based methods should be used to test whether changes in LL, SVA, PI–LL, PT, and related parameters predict clinically meaningful changes in ODI, VAS, satisfaction, and function. Long-term studies should also evaluate adjacent-segment degeneration, reoperation, correction loss, and mechanical failure. Prediction models may incorporate alignment, facet orientation, muscle quality, bone density, age, and surgical approach, but they require external validation before clinical use.

### 3.7 Conclusion

Two-level lumbar spondylolisthesis surgery generally produces greater regional lordotic correction than single-level fusion, but improvement in global sagittal balance and patient-reported outcomes is less uniform. LL, SVA, PT, SS, and PI–LL are clinically relevant because they help predict ODI and VAS MCID achievement, yet they should be interpreted as radiographic predictors and alignment targets rather than independent MCID outcomes. Current thresholds remain provisional because direct two-level evidence is limited and heterogeneous. Individualized planning based on pelvic morphology, baseline alignment, operated levels, age, bone quality, and paraspinal muscle condition offers the most defensible basis for surgical correction and postoperative evaluation.
