# Supplementary material for: Research progress on sagittal balance and minimal clinically important difference after two-level lumbar spondylolisthesis surgery
Source: Front Surg. 2026 Sep 1;13:1912779. doi: 10.3389/fsurg.2026.1912779 (PMC13575145; doi:10.3389/fsurg.2026.1912779)
Supplement: Supplementary file 1 [file Table1.docx]

**Table S1. Operational eligibility definitions**

| **Criterion** | **Operational definition** |
| --- | --- |
| Two-level lumbar spondylolisthesis | Vertebral translation at two lumbar motion segments or fusion across two interbody levels |
| Multilevel evidence | Two or more fused levels with separable two-level findings or direct relevance to two-level correction |
| Sagittal outcome | LL, SLL, SVA, PI, PT, SS, PI–LL, T1PA, GAP score, or lordosis distribution |
| MCID-related evidence | MCID for a validated patient-reported outcome or radiographic predictors of MCID achievement |
| Radiographic predictor | Sagittal variable associated with achievement of ODI, VAS, or another patient-reported MCID |
| Alignment target | Postoperative radiographic value proposed to guide correction without classification as MCID |
| Foundational article | Pre-2010 study defining or validating MCID, sagittal parameters, HRQoL associations, or alignment classifications |
| High risk of bias | NOS score ≤4, ROBIS high risk, or AMSTAR 2 critically low confidence |
| Incomplete evidence | Unavailable full text, absent methods, abstract-only report, or non-extractable outcomes |

**Table S2. Complete electronic search strategies**

| **Source** | **Complete search syntax** |
| --- | --- |
| PubMed/MEDLINE | (("Spondylolisthesis"[Mesh] OR "lumbar spondylolisthesis"[tiab] OR "degenerative spondylolisthesis"[tiab] OR "isthmic spondylolisthesis"[tiab] OR "double-level spondylolisthesis"[tiab] OR "two-level spondylolisthesis"[tiab] OR "multilevel spondylolisthesis"[tiab]) AND ("Spinal Fusion"[Mesh] OR "lumbar fusion"[tiab] OR "interbody fusion"[tiab] OR PLIF[tiab] OR TLIF[tiab] OR OLIF[tiab] OR ALIF[tiab] OR LLIF[tiab]) AND (("sagittal balance"[tiab] OR "sagittal alignment"[tiab] OR spinopelvic[tiab] OR "lumbar lordosis"[tiab] OR "segmental lordosis"[tiab] OR "sagittal vertical axis"[tiab] OR "pelvic incidence"[tiab] OR "pelvic tilt"[tiab] OR "sacral slope"[tiab] OR "PI-LL"[tiab] OR "T1 pelvic angle"[tiab] OR "global alignment and proportion"[tiab]) OR ("minimal clinically important difference"[tiab] OR MCID[tiab] OR "Oswestry Disability Index"[tiab] OR "visual analog scale"[tiab] OR "visual analogue scale"[tiab] OR "patient-reported outcome*"[tiab] OR "health-related quality of life"[tiab]))) AND ("2010/01/01"[Date - Publication] : "2026/03/31"[Date - Publication]) AND (english[lang] OR chinese[lang]) |
| Embase | ('lumbar spondylolisthesis'/exp OR 'degenerative spondylolisthesis':ti,ab,kw OR 'isthmic spondylolisthesis':ti,ab,kw OR 'two-level spondylolisthesis':ti,ab,kw OR 'double-level spondylolisthesis':ti,ab,kw OR 'multilevel spondylolisthesis':ti,ab,kw) AND ('spinal fusion'/exp OR 'lumbar fusion':ti,ab,kw OR 'interbody fusion':ti,ab,kw OR PLIF:ti,ab,kw OR TLIF:ti,ab,kw OR OLIF:ti,ab,kw OR ALIF:ti,ab,kw OR LLIF:ti,ab,kw) AND (('sagittal balance':ti,ab,kw OR 'sagittal alignment':ti,ab,kw OR spinopelvic:ti,ab,kw OR 'lumbar lordosis':ti,ab,kw OR 'segmental lordosis':ti,ab,kw OR 'sagittal vertical axis':ti,ab,kw OR 'pelvic incidence':ti,ab,kw OR 'pelvic tilt':ti,ab,kw OR 'sacral slope':ti,ab,kw OR 'PI-LL':ti,ab,kw OR 'T1 pelvic angle':ti,ab,kw OR 'global alignment and proportion':ti,ab,kw) OR ('minimal clinically important difference':ti,ab,kw OR MCID:ti,ab,kw OR 'Oswestry Disability Index':ti,ab,kw OR 'visual analog scale':ti,ab,kw OR 'patient reported outcome':ti,ab,kw OR 'health related quality of life':ti,ab,kw)) AND [2010-2026]/py AND ([english]/lim OR [chinese]/lim) |
| Scopus | TITLE-ABS-KEY(("lumbar spondylolisthesis" OR "degenerative spondylolisthesis" OR "isthmic spondylolisthesis" OR "two-level spondylolisthesis" OR "double-level spondylolisthesis" OR "multilevel spondylolisthesis") AND ("lumbar fusion" OR "interbody fusion" OR PLIF OR TLIF OR OLIF OR ALIF OR LLIF) AND (("sagittal balance" OR "sagittal alignment" OR spinopelvic OR "lumbar lordosis" OR "segmental lordosis" OR "sagittal vertical axis" OR "pelvic incidence" OR "pelvic tilt" OR "sacral slope" OR "PI-LL" OR "T1 pelvic angle" OR "global alignment and proportion") OR ("minimal clinically important difference" OR MCID OR "Oswestry Disability Index" OR "visual analog scale" OR "patient-reported outcome" OR "health-related quality of life"))) AND PUBYEAR > 2009 AND PUBYEAR < 2027 |
| Web of Science Core Collection | TS=(("lumbar spondylolisthesis" OR "degenerative spondylolisthesis" OR "isthmic spondylolisthesis" OR "two-level spondylolisthesis" OR "double-level spondylolisthesis" OR "multilevel spondylolisthesis") AND ("lumbar fusion" OR "interbody fusion" OR PLIF OR TLIF OR OLIF OR ALIF OR LLIF) AND (("sagittal balance" OR "sagittal alignment" OR spinopelvic OR "lumbar lordosis" OR "segmental lordosis" OR "sagittal vertical axis" OR "pelvic incidence" OR "pelvic tilt" OR "sacral slope" OR "PI-LL" OR "T1 pelvic angle" OR GAP) OR ("minimal clinically important difference" OR MCID OR ODI OR VAS OR "patient-reported outcome" OR "health-related quality of life"))) |
| Cochrane Library | ([mh Spondylolisthesis] OR "lumbar spondylolisthesis":ti,ab,kw OR "degenerative spondylolisthesis":ti,ab,kw) AND ([mh "Spinal Fusion"] OR "lumbar fusion":ti,ab,kw OR "interbody fusion":ti,ab,kw) AND ("sagittal balance":ti,ab,kw OR "lumbar lordosis":ti,ab,kw OR "sagittal vertical axis":ti,ab,kw OR "pelvic incidence":ti,ab,kw OR "minimal clinically important difference":ti,ab,kw OR MCID:ti,ab,kw OR ODI:ti,ab,kw) |
| ScienceDirect | ("lumbar spondylolisthesis" OR "degenerative spondylolisthesis") AND ("two-level" OR "double-level" OR multilevel OR fusion) AND ("sagittal balance" OR "lumbar lordosis" OR "PI-LL" OR "sagittal vertical axis" OR MCID OR "minimal clinically important difference" OR ODI) |
| SpringerLink | ("lumbar spondylolisthesis" AND ("two-level" OR "double-level" OR multilevel) AND ("sagittal alignment" OR "spinopelvic parameters" OR "lumbar lordosis" OR "PI-LL" OR MCID OR "patient-reported outcome")) |
| CNKI | 主题=("腰椎滑脱" OR "退行性腰椎滑脱" OR "峡部裂性腰椎滑脱") AND 主题=("双节段" OR "两节段" OR "多节段" OR "腰椎融合") AND 主题=("矢状位平衡" OR "脊柱骨盆参数" OR "腰椎前凸" OR "矢状位垂直轴" OR "骨盆入射角" OR "骨盆倾斜角" OR "骶骨倾斜角" OR "最小临床重要差异" OR "Oswestry功能障碍指数") |
| Wanfang Data | ("腰椎滑脱" OR "退行性腰椎滑脱") AND ("双节段" OR "两节段" OR "多节段融合") AND ("矢状位平衡" OR "腰椎前凸" OR "脊柱骨盆参数" OR "PI-LL" OR "最小临床重要差异" OR "ODI") |
| VIP Database | M=("腰椎滑脱" OR "退行性腰椎滑脱") AND M=("双节段" OR "两节段" OR "多节段") AND M=("腰椎融合" OR "椎间融合") AND M=("矢状位平衡" OR "腰椎前凸" OR "脊柱骨盆参数" OR "最小临床重要差异") |

**Table S3. Full-text exclusions**

| **Exclusion reason** | **Number** |
| --- | --- |
| Single-level population without a two-level comparator or separable subgroup | 52 |
| No sagittal or MCID-related outcome | 39 |
| Irrelevant or non-surgical intervention | 21 |
| Commentary, technical note, or non-systematic review | 14 |
| Duplicate or overlapping cohort | 11 |
| Insufficient methods or outcome data | 9 |
| Conference abstract without full report | 5 |
| Language other than English or Chinese | 5 |
| **Total** | **156** |

**Table S4. Standardized data-extraction fields**

| **Domain** | **Extracted variables** |
| --- | --- |
| Study identification | Author, year, country, journal, language, recruitment period |
| Design and setting | Study design, centre number, hospital type |
| Population | Sample size, age, sex, body mass index, diagnosis, slip grade |
| Disease distribution | Operated levels, segment combination, pathology, comparator |
| Surgery | Approach, open or minimally invasive technique, cage type and angle |
| Follow-up | Minimum, mean, and final duration |
| Sagittal outcomes | LL, SLL, SVA, PI, PT, SS, PI–LL, T1PA, GAP score |
| Additional imaging outcomes | Disc height, slip percentage, reduction, fusion, adjacent-segment degeneration |
| Clinical outcomes | ODI, VAS, SF-12, SF-36, SRS-22, JOA, satisfaction |
| MCID method | Instrument, anchor, calculation method, threshold, follow-up, achievement rate |
| Predictor analysis | Model, covariates, effect estimate, confidence interval, discrimination |
| Quality indicators | Selection, confounding, attrition, reporting, funding, conflicts |

**Table S5. Quality-appraisal framework**

| **Evidence type** | **Tool** | **Domains** | **Interpretation** |
| --- | --- | --- | --- |
| Cohort and case-control studies | NOS | Selection, comparability, outcome or exposure | 7–9 low risk; 5–6 moderate risk; 0–4 high risk |
| Systematic reviews and meta-analyses | AMSTAR 2 | Protocol, search, selection, appraisal, synthesis, publication bias | High, moderate, low, or critically low confidence |
| Systematic reviews and meta-analyses | ROBIS | Eligibility, identification, appraisal, synthesis | Low, high, or unclear risk |
| Foundational studies | Conceptual-validation criteria | Definition, derivation, reproducibility, external use | Contextual evidence only |
